# Tuning light emission of PbS nanocrystals from infrared to visible range by cation exchange

**DOI:** 10.1088/1468-6996/16/5/055007

**Published:** 2015-10-27

**Authors:** Enrico Binetti, Marinella Striccoli, Teresa Sibillano, Cinzia Giannini, Rosaria Brescia, Andrea Falqui, Roberto Comparelli, Michela Corricelli, Raffaele Tommasi, Angela Agostiano, M Lucia Curri

**Affiliations:** 1 CNR-IPCF Div. Bari, c/o Department of Chemistry, via Orabona 4, 70126 Bari, Italy; 2 CNR- IC Institute of Crystallography, National Research Council, Via Amendola 122/O, 70126 Bari, Italy; 3 Nanochemistry Department, Istituto Italiano di Tecnologia, via Morego 30, 16163 Genova, Italy; 4 Biological and Environmental Sciences and Engineering Division, King Abdullah University of Science and Technology (KAUST), 23955-6900 Thuwal, Kingdom of Saudi Arabia; 5 Department of Chemistry, University of Bari Aldo Moro, Via Orabona 4, 70126 Bari, Italy; 6 Department of Basic Medical Sciences, Neuroscience and Sense Organs, University of Bari Aldo Moro, Piazza G. Cesare 11, 70124 Bari, Italy

**Keywords:** colloidal nanocrystals, cation exchange, PbS, luminescent materials

## Abstract

Colloidal semiconductor nanocrystals, with intense and sharp-line emission between red and near-infrared spectral regions, are of great interest for optoelectronic and bio-imaging applications. The growth of an inorganic passivation layer on nanocrystal surfaces is a common strategy to improve their chemical and optical stability and their photoluminescence quantum yield. In particular, cation exchange is a suitable approach for shell growth at the expense of the nanocrystal core size. Here, the cation exchange process is used to promote the formation of a CdS passivation layer on the surface of very small PbS nanocrystals (2.3 nm in diameter), blue shifting their optical spectra and yielding luminescent and stable nanostructures emitting in the range of 700–850 nm. Structural, morphological and compositional investigation confirms the nanocrystal size contraction after the cation-exchange process, while the PbS rock-salt crystalline phase is retained. Absorption and photoluminescence spectroscopy demonstrate the growth of a passivation layer with a decrease of the PbS core size, as inferred by the blue-shift of the excitonic peaks. The surface passivation strongly increases the photoluminescence intensity and the excited state lifetime. In addition, the nanocrystals reveal increased stability against oxidation over time. Thanks to their absorption and emission spectral range and the slow recombination dynamics, such highly luminescent nano-objects can find interesting applications in sensitized photovoltaic cells and light-emitting devices.

## 1. Introduction

Quantum confinement in semiconductor nanocrystals (NCs) modifies the electronic structure of the bulk materials and offers the possibility to tune their optical and electrical properties merely by varying the NC size and shape [1]. Among the different synthetic approaches available for the growth of semiconductor NCs, colloidal synthesis is an inexpensive route that guarantees precise control over NC size and shape. Colloidal NCs are free-standing nanometer sized objects (1–10 nm in diameter), coated with a layer of organic capping ligands which allows their dispersion in solvents, making manipulation easy [2]. Organic ligands play the essential role in controlling the NC surface during all synthesis phases and preventing aggregation phenomena, thus keeping their size-distribution narrow [3]. In addition, colloidal NCs can be further exposed to a wide variety of post-synthetic processes to improve their stability and/or their performance [4–7]. Indeed they have been successfully applied in optoelectronics, such as in light emitting diodes (LEDs), solar cells and photo-detectors, as well as in biomedical labeling applications, both *in vivo* and *in vitro* [8–12].

The majority of applications are based on the size-dependent optical properties of semiconductor NCs, generally characterized by a band-edge absorption peak, which redshifts with the increase of NC size and a wide-ranging absorption at higher energy [13]. Their high extinction coefficient and large absorption band play a central role in energy conversion devices, where NCs are generally used to efficiently absorb solar radiation, allowing the production of electric energy [14]. On the other hand, semiconductor NCs can also exhibit an intense and sharp-line photoluminescence, which is appealing for the fabrication of pure-color light emitting devices [15]. However, the elevated surface/volume ratio and high surface defect density induce the formation of intra-gap trap states, which lead to non-radiative relaxation processes [16]. Organic ligands allow the stabilization of the surface atoms, forming complexes, but such passivation is often not stable and uniform [17]. Therefore, an effective surface passivation and subsequent enhancement of the linear optical properties (i.e. photoluminescence (PL) emission, PL quantum yield, and excited-states lifetime) are achieved by growing an inorganic shell of a wider band-gap semiconductor on the NC surface [17]. In such a case, the inorganic shell provides a physical barrier between the core and the surrounding medium, thus making NCs less sensitive to environmental effects, surface chemistry and photo-oxidation [18, 19]. Furthermore, such a shell can effectively passivate surface defects (trap sites) and further concentrate charge carriers in the NC core, far from the NC surface, leading to a reduction of non-radiative losses and, consequently, to an enhancement of radiative recombination processes [20].

Nowadays, controlling the NC size (<10 nm) and tuning the stoichiometry (from ZnCdS–ZnS core–shell NCs to CdZnSe NCs) [21], the PL emission of II–VI semiconductor NCs fully covers the visible spectrum from blue to red, with different quantum efficiencies. Colloidal I−III−VI NCs, such as CuInS_2_ and AgInS_2_, have also been investigated in light-emitting and solar applications, as safe and non-toxic materials [22]. The extension of the spectroscopic properties in the near IR up to mid-IR has been achieved with lead chalcogenides (PbS, PbSe and PbTe NCs) and with CdHgTe and InAs based NCs, which have shown promising performance as emitters [23–27]. The spectral region between the visible and NIR is of particular interest due to the transparency windows of blood and tissue, which reduce absorption and auto-fluorescence, and are thus particularly suited for bioimaging applications [28].

However, while the largest NCs based on Cd-chalcogenides efficiently emit up to 650 nm, the smallest Pb-chalcogenide NCs start to emit above 850 nm, leaving the wavelength window between these values uncovered [29–31]. Furthermore, lead chalcogenide NCs are highly unstable under ambient conditions, due to the destructive and irreversible oxidative processes occurring at the NC surface [32]. Such NC oxidation can be detected as a quenching and blue-shift of the PL peak due to the size-reduction caused by the formation of oxides at the NC surface.

The stability against oxidation in air has been achieved with inorganic shells made of air-stable materials, such as CdS or CdSe coated with a further stabilizing shell of ZnS [33]. Several authors reported the growth of the shell by exposure of NCs to both organometallic precursors of the shell materials, such as Zn and S precursors for a ZnS shell [15]. Recently core–shell NCs have been synthesized via a cation exchange synthetic route [33–35]. In particular, NCs are exposed to a large excess of one precursor of the materials constituting the shell, which grows at the expense of the NCs, i.e. accompanied by the decrease of the core diameter [36]. One of the main advantages of this process consists in its careful control, which allows for the formation of homogeneous alloys [37], gradient concentrated alloys [38], doped NCs [39] and core–shell heterostructures [40]. In addition, the cation exchange approach enables the complete conversion of the chemical composition of colloidal NCs, making possible the fabrication and control over size and shape of nanomaterials that would be otherwise hard to synthesize through a direct reaction [41].

The cation-exchange procedure has also been used not only for the synthesis of core–shell NCs, such as Se@ZnSe, Se@CdSe, Se@PbSe, PbS@CdS and PbSe@CdSe [33, 36], but also for the preparation of seeds that could be used for the successive growth of branched cadmium chalcogenide NCs [42]. Recently, as an alternative to the cation exchange approach, anion exchange has been carried out on cesium lead halide perovskite NCs, resulting in NCs with tunable optical properties over the entire visible spectral region [43, 44].

Here we report on the colloidal synthesis, cation exchange process, structural and spectroscopic characterization of very small PbS–CdS NCs, brightly emitting in the spectral region between red and NIR (700–900 nm). First, very small PbS NCs (2.3 nm in diameter) were synthesized by pyrolysis of organometallic precursors injected in a suitable mixture of hot organic coordinating solvents [45, 46]. Subsequently, PbS–CdS NCs were prepared by Pb to Cd cation exchange at the surface of the pre-synthesized PbS NCs, following a synthetic strategy already demonstrated to be successful for the growth of a CdS shell on large PbS NCs [47]. Synthesized PbS–CdS NCs were characterized during the cation exchange process by steady-state and time-resolved optical spectroscopies, transmission electron microscopy (TEM), x-ray powder diffraction (XRPD), high-resolution TEM (HR-TEM) and energy-dispersive x-ray spectroscopy in scanning TEM. The reduction of the core diameter was confirmed by morphologic and diffraction techniques (TEM, HR-TEM and XRPD), which demonstrated a lattice contraction of the external PbS layer after cation exchange and a size reduction of the NCs with respect to the native PbS. The spectroscopic characterization indicated that the electronic transitions, responsible for the optical emission, are compatible with a reduction of the PbS core, although STEM-EDS mapping has not allowed us to clearly assess the formation and shell composition in such small NCs. In addition, the recombination dynamics confirmed the effective surface passivation, as also supported by the strong enhancement of the PL emission and the increased optical stability over time.

## 2. Experimental

### 2.1. Materials

PbO (99.999%), CdO (99.5%), oleic acid (OLEA, 90%), trioctylphosphine, (TOP, 90%), 1-octadecene (ODE, 90%), hexamethyldisilathiane (HMDS, synthesis grade), anhydrous toluene (99.8%), isopropanol (99.8%) and acetone (99.5%) were purchased from Sigma Aldrich. Ethanol (99.8%) was purchased from Fluka.

### 2.2. Synthesis of PbS–CdS NCs

PbS–CdS core–shell NCs were prepared by performing a Pb to Cd cation exchange on synthesized and purified PbS NCs. The synthesis of PbS NCs was carried out in a three-neck flask connected to a Schlenk line. 0.9 g (4 mmol) of PbO, 2.0 g (7 mmol) of OLEA and 36 mL of ODE were heated at 150 °C up to the complete decomposition of PbO in Pb–oleate moieties. Such a mixture was dried under vacuum at 150 °C for 30 min, and 3 mL (6.7 mmol) of TOP was injected with a syringe. After 2 min the flask was poured under N_2_ flow, and a solution of 0.420 mL (2 mmol) of HMDS in 4 mL of anhydrous ODE was quickly injected at 110 °C, and the temperature was left to decrease down to 30 °C. The synthesis product was washed with a mixture of ethanol, acetone and isopropanol by centrifugation (3000 rpm, 10 min), yielding approximately 480 mg of PbS NCs. The synthesis product was dried under vacuum and dispersed in 10 mL of anhydrous toluene in a glovebox.

**Figure 1.**
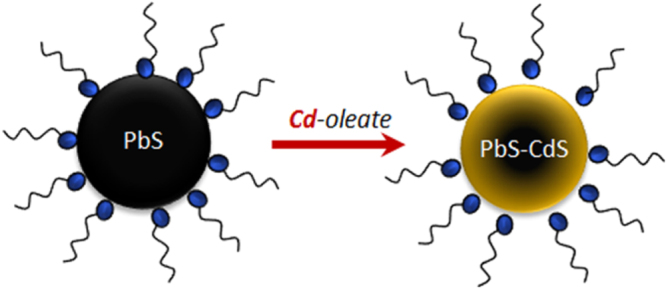
Scheme of the Pb to Cd cation exchange on the PbS NC surface.

For the CdS shell synthesis, 0.44 g (3.4 mmol) of CdO, 2.4 g (8.5 mmol) of OLEA and 40 mL of ODE were placed in a three-neck flask. The mixture was heated up to 220 °C under air up to the complete decomposition of CdO, and was then dried at 150 °C under vacuum for about 1 h. At this point the flask was re-opened to a N_2_ flow, and 5 mL of the starting solution of PbS NCs was injected at *T* = 70 °C. Synthesis aliquots were taken after 30 and 180 seconds (s), and cooled with a cold non-solvent mixture (ethanol:acetone:isopropanol 1:1:1). After 300 s the product was cooled with the same cold mixture. All the products were then precipitated by centrifugation (3000 rpm, 10 min), dried under vacuum and dispersed in 5 mL of anhydrous toluene in a glovebox. The scheme of the Pb to Cd cation exchange procedure is reported in figure 1. PbS and PbS–CdS NCs were characterized by XRPD, TEM, HR-TEM and STEM-EDS, while their optical properties were investigated by optical absorption and steady state and time-resolved PL spectroscopy.

### 2.3. Structural and compositional characterizations of NCs

XRPD patterns were collected at room temperature from NCs deposited onto miscut silicon substrates. Measurements were performed in coupled sample/detector (*θ*/2*θ*) scan mode (symmetric reflection geometry) by a Bruker D8 Discover diffractometer, equipped with a Göbel mirror, using Cu Kα radiation (*λ*K*α*1 = 1.540 56 Å and *λ*K*α*2 = 1.544 39 Å), and a scintillation detector. The working conditions were 40 kV and 40 mA. Data were collected in the range 15–100° with a step size of 0.05°.

For TEM analyses, the samples were prepared by drop-casting 1 *μ*L of the NC suspension onto a Cu grid coated with an ultrathin carbon film. Overview TEM images and selected-area electron diffraction (SAED) patterns were recorded on a JEOL JEM-1011 TEM (100 kV), the latter by selecting areas (∼1 *μ*m diameter) including several NCs and calibrated based on a SAED pattern acquired in identical conditions on a nanocrystalline Au film deposited onto a carbon coated grid. Beam-stop removal, azimuthal integration and background subtraction were carried out using the PASAD-tools (v 0.1) plugin for Gatan Digital Micrograph [48].

HR-TEM and EDS analyses, performed in STEM mode, were carried out on a JEOL JEM-2200FS microscope operated at 200 kV, equipped with a CEOS image aberration corrector and a Bruker Quantax 400 system with a 60 mm^2^ silicon-drift detector (SDD). For EDS quantification the Cliff–Lorimer method, implemented in the Esprit software, was used for the Cd L, Pb L and S K peak series. Given the small NC size, and consequently the low signal-to-noise ratio in the EDS spectra, and the peak deconvolution needed for S quantification, an uncertainty between 5% and 11% was estimated on the evaluation of the extracted element percentage.

### 2.4. Spectroscopic characterizations of NCs

Optical absorption spectra were recorded with a Cary 5000 (Varian) spectrophotometer. PL emission spectra were recorded by using a HORIBA Jobin-Yvon Fluorolog 3 spectrofluorimeter, equipped with a 450 W Xe-lamp and double grating excitation and emission monochromators. Time-resolved PL measurements were performed by using the time-correlated single photon counting (TCSPC) technique, with an HORIBA Jobin-Yvon FluoroHub. The experimental setup was equipped with a laser diode (NanoLED 375L) emitting ≈80 ps pulses at 375 nm with a repetition rate tunable between 10 kHz and 100 MHz. The PL was dispersed by a double grating monochromator and detected by a picosecond photon counter (TBX ps Photon Detection Module, HORIBA Jobin-Yvon) with a temporal resolution of about 200 ps. The average decay lifetime was calculated as reported in the supplementary data.

## 3. Results and discussion

**Figure 2.**
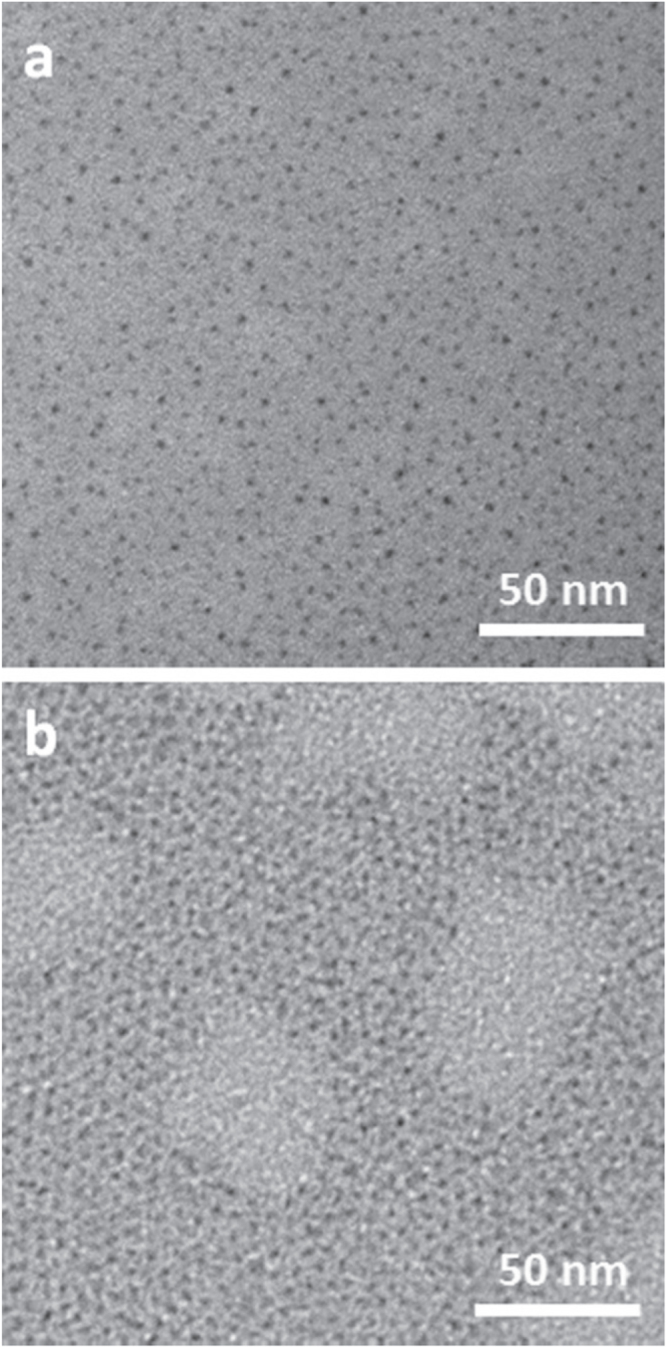
TEM images of (a) PbS NCs and (b) PbS–CdS NCs.

Colloidal PbS NCs, 2.3 nm in size, were synthesized according to the procedure described in the experimental section and subsequently subjected to the Pb to Cd cation exchange process. As the pristine PbS NCs, the PbS–CdS NCs are capped with OLEA and easily dispersible in apolar solvents (i.e. toluene). The morphology of the synthesized NCs was preliminarily investigated by low-resolution TEM measurements, performed by depositing NCs directly on TEM grids; the micrographs are reported in figure 2. At first glance, TEM images do not reveal significant modifications in NC morphology after the cationic exchange process and both PbS and PbS–CdS NCs appear, at first approximation, spherical in shape. In addition, no aggregation was observed, due to the long alkyl chain of the capping agent. However, for such small NCs the low resolution TEM did not allow us to measure accurately neither size nor size-distribution.

**Figure 3.**
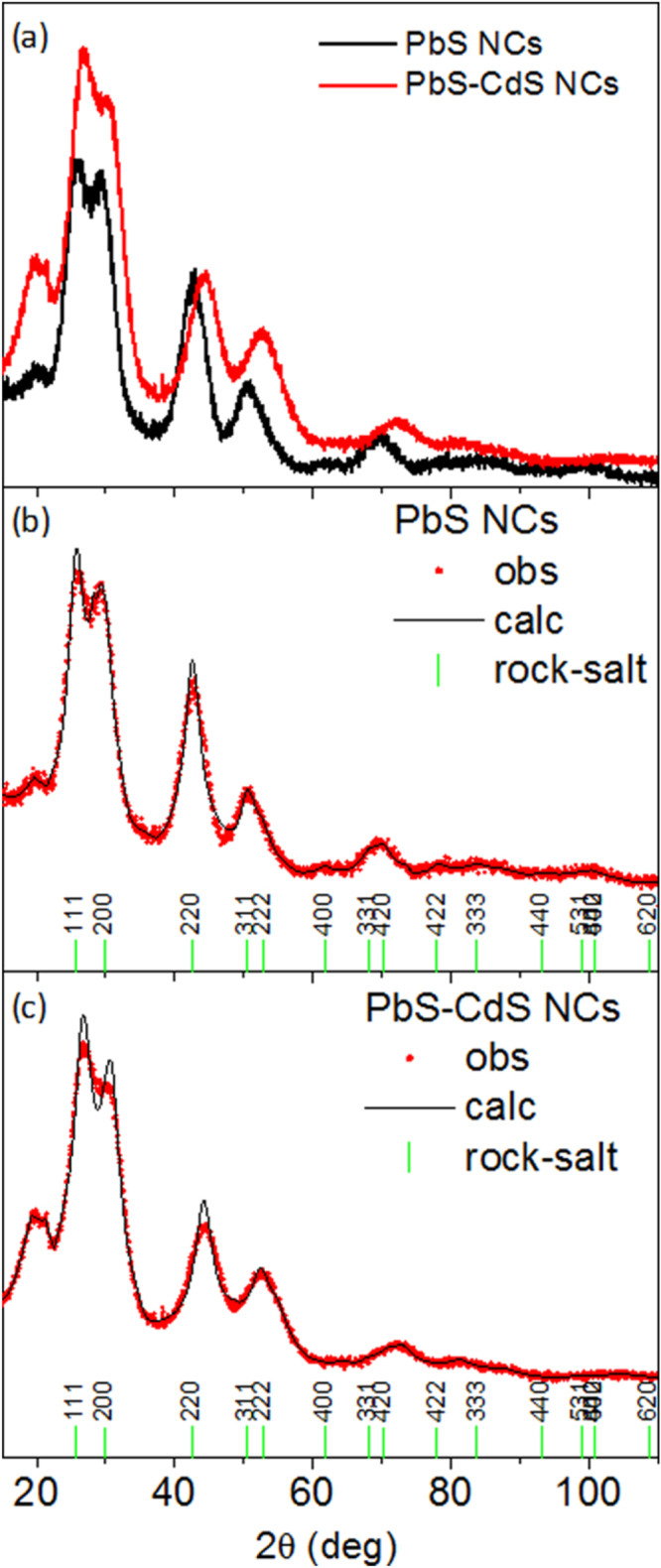
(a) XRPD patterns for PbS (black curve) and PbS–CdS (red curve) NCs; (b) Rietveld refinement XRPD pattern from PbS NCs; (c) Rietveld refinement XRPD pattern from PbS–CdS NCs. Red circles: XRD experimental data; black line: calculated powder pattern; vertical bars: Bragg reflections positions of the PbS crystal structure.

In order to estimate the diameter of the nanoparticles and the main change in the crystalline structure of the expected PbS–CdS core–shell NCs with respect to the pre-synthesized PbS NCs, XRPD analysis was carried out. In case of large PbS–CdS core–shell systems, recent literature reports a rock salt crystal structure for the PbS core, while a zinc blend structure is measured for the thick CdS shell [49]. Figure 3(a) shows a comparison between the collected data for the PbS core (black curve) and PbS–CdS (red curve) NCs. Two major differences can be clearly detected: (i) the Bragg reflections of the PbS–CdS XRPD pattern are positioned at higher 2*θ* scattering angles, with respect to the same reflections of the PbS XRPD spectrum; (ii) the width of the diffraction peaks for the PbS–CdS curve are larger with respect to the PbS curve. A whole profile XRPD fitting was performed using Fullprof software [50].

The instrumental resolution function (IRF) was evaluated by fitting the XRPD pattern collected on a LaB6 NIST standard, measured in the same experimental conditions. The agreement between the observed and calculated XRPD patterns of PbS and PbS–CdS NCs, as obtained by the Rietveld refinement, are pictured in figures 3(b) and (c), respectively.

In our case both patterns can be indexed as cubic PbS rock-salt structure (ISCD # 38293), but the Bragg reflections positioned at higher 2*θ*, in figure 3(c), can be justified by a contraction of the a-lattice parameter of approximately 4% in the PbS–CdS NCs. Furthermore, the whole profile fitting analysis of the spectra indicates a slight decrease in the apparent size of the PbS core from 2.4 ± 0.5 nm, estimated from the PbS NCs, to 2.0 ± 0.5 nm. The contraction of the lattice constant, measured in the PbS–CdS NC system, can be explained in two ways: either as lattice contraction due to the statistical substitution of guest ions in the host lattice, in accordance with the predictions of Vegard’s law [51, 52], or as a strong contraction effect due to the surface strain field. For the first explanation, statistical substitutions of Cd ions in the PbS host lattice are expected to produce a lattice contraction—as found from the XRPD analysis—because of the smaller ionic radius Cd with respect to Pb [53, 54]. In the case of an extremely high surface effect, it is worth recalling that the smaller the nanoparticle size the highest the surface-to-volume ratio [55]. At a size of 2.0–2.5 nm, as for the NCs studied here, the surface (shell) is still expected to have quite a significant effect on the volume, i.e. on the core atom arrangements, causing important strain effects on the lattice (see the supplementary data). On the other hand, the formation of ternary Cd_1−*x*_Pb*_x_*S structures, by exchanging Pb and Cd cations, can be excluded since PbS and CdS are not miscible at low temperature [35, 49].

**Figure 4.**
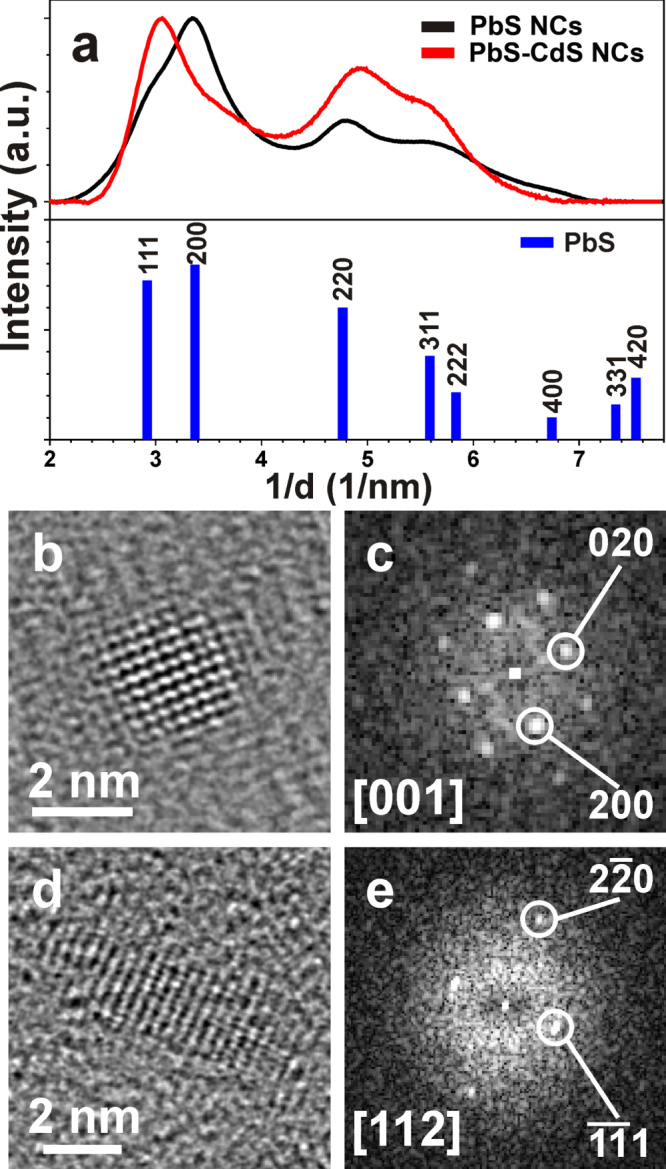
(a) Background-subtracted, azimuthally integrated SAED patterns obtained on the PbS (black curve) and PbS–CdS (red curve) NC samples, compared with Bragg reflections positions for cubic PbS rock-salt structure (ICSD # 38293). (b), (d) HR-TEM images and (c), (e) corresponding fast Fourier transforms (FT) of single NCs in the (b) PbS and (d) PbS–CdS samples, compatible with cubic structures with lattice constants (b), (c) *a* = 6.4 Å and (d), (e) *a* = 5.6 Å, respectively. An HR-TEM image and corresponding FT of a relatively large PbS NC (6.9 nm Feret diameter) is reported as figure S2 of the supplementary data.

To investigate the NC composition, a quantitative STEM-EDS analysis was carried out on both PbS and PbS–CdS NCs. The STEM-EDS atomic quantification performed on a large number of PbS NCs shows an initial cation excess (Pb:S = 2:1), in agreement with the small size and cation-enriched surface observed for NCs prepared in similar conditions [56, 57]. After the cation exchange step, the overall composition evolves to Pb:Cd:S = 21:39:40, with a decreased cation excess ((Pb + Cd)/S = 1.5). EDS elemental mapping was not possible due to the very small size of the NCs, therefore hindering the confirmation of a possible core–shell structure or, alternatively, of a graded shell distribution of the cations within the NCs. In agreement with XRPD results, SAED data, collected by selecting a large number of NCs in both samples (figure 4(a)), exhibit a pattern from a cubic PbS rock-salt structure (in agreement with the ISCD # 38293), with a 5% contraction of the a-lattice parameter, going from 5.97 Å to 5.66 Å. HR-TEM analysis of single NCs, accordingly, show a lattice contraction upon cation exchange (figures 4(b)–(e)). Given the low number of cells within single NCs, the strain mapping does not allow us to evaluate mean dilatation, caused by a possible lattice contraction in the peripheral region due to CdS growth. It is worth noting that HR-TEM measurements, performed on larger NCs, evidence a modification of the NC diameter (intended as the Feret diameter, namely the projection of a three-dimensional object on a 2D plane) from 3.8(±1.0) nm to 2.8(±1.1) nm after cation exchange. Such a size and size-reduction seem to be in contrast with the values estimated by XRPD findings. However, the different measured NC diameters can be easily attributed to the consideration that the HR-TEM measurement was performed on larger NCs and the complementary information that can be deduced from the two investigation techniques. In fact, while HR-TEM analysis accounts for the largest internal diameter of the single-crystal particles clearly imaged (the larger ones), XRPD measures only the mean size of the diffracting domains of the whole NC population, not also taking into consideration the highly disordered external portion constituting the surface of such small NCs. The concomitance of these effects can explain the high standard deviation measured by TEM for the NC mean size, as well as the slight discrepancy between them and those indirectly extracted by XRPD.

**Figure 5.**
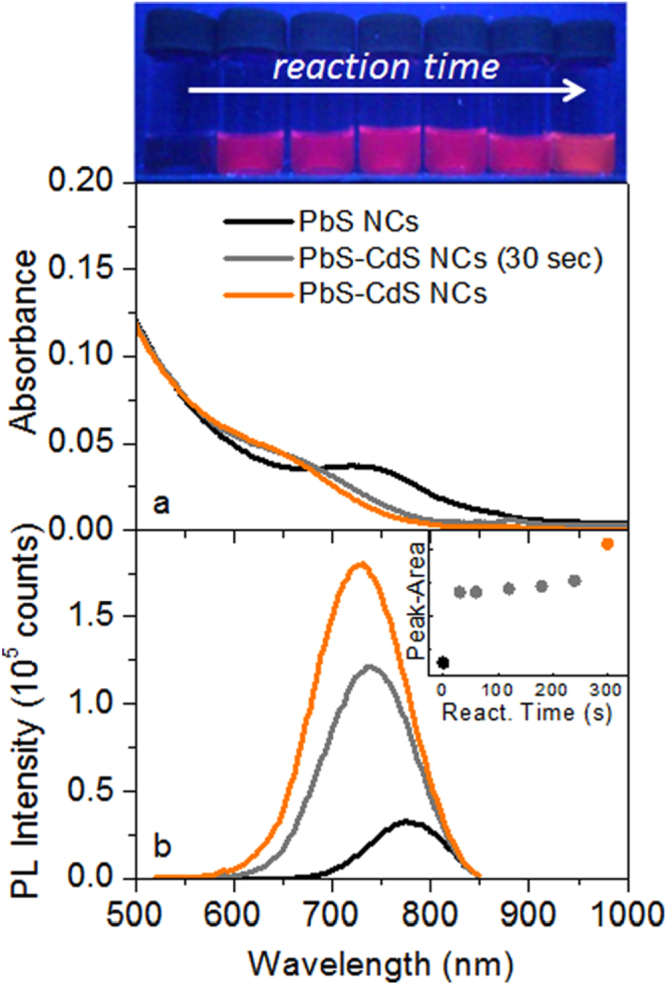
(a) Absorption and (a) PL spectra of PbS NCs, PbS–CdS NCs at 30 s of reaction time, and final PbS–CdS NCs. Inset: PL peak integrated area of the cation-exchanged samples at increasing reaction times (0 s indicates the native PbS NCs and 300 s the final purified PbS–CdS NCs). Top: pictures of the vials containing PbS NC samples, withdrawn at different times during the cation exchange process, under UV light. A detailed overview of the absorption and PL spectra is reported in the supplementary data.

The effect of the Pb to Cd cation exchange on the NC optical properties was also investigated by spectroscopic techniques. Figure 5(a) reports the absorption spectra of pristine PbS NCs and PbS–CdS NCs during the shell growth. The absorption line-shape of the PbS NCs clearly shows a band-edge peak at 730 nm, correlated to the original NC diameter and high-energy absorption contribution, ascribed to the continuum of energy states of semiconductors.

The excitonic absorption peak of PbS NCs becomes less evident during the cation exchange process, leading to blue-shifted absorption spectra, also for the shortest reaction time. At a reaction time of only 30 s, the band-edge absorption peak is no longer clearly visible, and the spectrum is overall blue-shifted with respect to bare PbS NCs. For longer reaction times, the absorption spectra remain unvaried, showing merely a slight progressive blue-shift (see figure S3 of the supplementary data). The sudden weakening of the PbS excitonic peak attests that the kinetic of surface cation exchange, under the experimental condition, is very fast. In agreement with the work of Justo *et al* [58], it is possible to use the PbS sizing curve to estimate the diameter of the PbS core in case of PbS–CdS core–shell nanostructures.

However, even if a true core–shell structure was not demonstrated by structural investigations due to the small NC size, the performed calculation of the PbS diameter in PbS–CdS NCs provide a value of 2.0 nm, in agreement with XRPD data.

Moreover, the decrease in intensity of the band-edge peak in the absorption spectra can be ascribed to both a size-distribution broadening of the PbS–CdS NCs and to a partial overlapping of the excitonic signal with the absorption component at higher energy, induced by the core diameter reduction [59]. The possible enlargement in size-distribution can be consistent with a cation exchange process occurring not uniformly at the NC surface.

Figure 5(b) reports the PL spectra of toluene dispersions of pristine PbS NCs and PbS–CdS NCs during the cation exchange reaction. It is worth noting that, in order to quantitatively compare the PL spectra, all NC solutions were diluted to obtain the same absorbance value at the excitation wavelength (*λ* = 500 nm). PbS NCs show a weak PL peak at 775 nm with a full width at half maximum (FWHM) of 90 nm, while, after only 30 s of reaction during the cation exchange process, PbS–CdS NCs exhibit a blue-shifted and more intense PL peak at 732 nm, with a FWHM of 110 nm. The PL intensity of PbS–CdS NCs slightly increases with the reaction time, while the peak position and FWHM remain almost unvaried. Such results can be rationalized attributing the emission to the presence of a PbS portion in the PbS–CdS NCs, smaller than native PbS NCs, thus with higher energy-gap [47]. The broadening of the emission line-shape can be correlated to the wider size distribution of PbS–CdS NCs, as already evidenced from absorption spectra. Longer reaction times do not further increase the PL intensity (see figure S4 of the supplementary data), thus demonstrating that the cation exchange reaches a rapid saturation after only 300 s. PL spectra of the NCs having the same absorbance at the excitation wavelength (500 nm), recorded under the same experimental conditions, can be reasonably compared. In our case the PL peak area of PbS–CdS NCs was 6.9 times greater than the area under the PL peak of as-synthesized PbS NCs. Such a large enhancement, directly related to the PL quantum yield, is ascribable to an efficient passivation of the surface trap-states induced by the cation exchange process [20].

**Figure 6.**
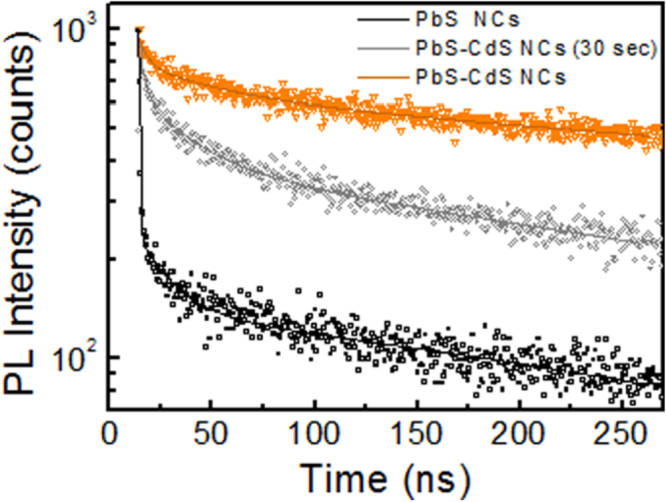
PL decays of PbS NCs and PbS–CdS NCs after 30 s of reaction time and at the end.

Time-resolved PL measurements were carried out to examine the effect of the CdS passivation layer on PbS NCs in terms of excited state lifetime. In figure 6 the PL decays acquired for PbS NCs and PbS–CdS NCs are reported. As expected, PbS NCs exhibited a recombination lifetime of the order of hundreds of nanoseconds, as already demonstrated for lead chalcogenide NCs [25]. In agreement with the PL spectra (figure 5), PbS NCs show the fastest PL decay, ascribed to the high number of surface defects that feed non-radiative recombination processes [20, 25]. A progressive slow-down of decays was observed during the cation exchange process, in agreement with the PL enhancement, further proving an effective passivation of PbS surface defects. PL decays properly interpolate by a three-exponential function, in good agreement with experimental data. The cation exchange significantly affects the first 10–20 ns of the decay, which is notably faster in PbS NCs than in PbS–CdS NCs, while the decays have a similar slope at longer times. The calculated average lifetimes, reported in table 1 (see the supplementary data for details), increase with the exchange process, from 424 ns for PbS NCs to 742 ns for PbS–CdS NCs at 300 s. In addition, it is worth noting that PbS–CdS heterostructures exhibit an accentuated valence-band offset but a small conduction-band offset. Therefore, while electron wave functions can extend into the CdS shell, holes are efficiently confined in the PbS core, limiting the wave function overlap (i.e. the electron-hole recombination probability) at the core edge [60, 61].

**Table 1.** Fitting parameters of PL decays of PbS NCs and PbS–CdS NCs. The weight coefficients are reported in square brackets. Further details are available in the supplementary data.

Sample	*t*_1_ (ns)	*t*_2_ (ns)	*t*_3_ (ns)	*t*_avg_ (*μ*s)	*χ*_sq_
PbS	0.25 [3.5]	10.4 [2.1]	424.3 [94.4]	424.1	1.06
PbS–CdS	0.55 [18.2]	20.5 [0.8]	742.0 [80.9]	741.7	1.15

**Figure 7.**
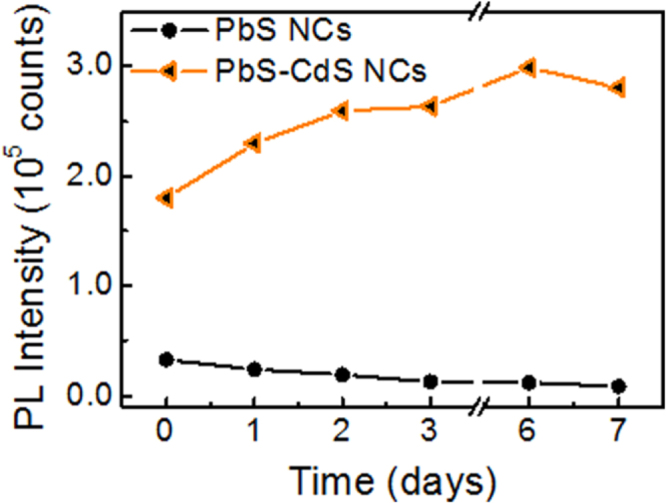
Temporal evolution of the PL peak intensity of PbS and PbS–CdS NCs. (The corresponding spectra are reported in figure S5 of the supplementary data.)

The PL stability of both PbS and PbS–CdS NCs was monitored daily over 7 days. Figure 7 reports the temporal evolution of the PL peak intensities measured for PbS NCs and PbS–CdS NCs. No significant alterations of the PL line-shape were detected (see figure S5 of the supplementary data) in the investigated temporal window, while the PL intensity of PbS NCs progressively decreases, up to 25% of the starting value after 7 days. Such behavior can be ascribed to a surface oxidation process, which affects the PL of PbS NCs. In contrast, the PL intensity of PbS–CdS NCs continues to increase up to 150% of the starting value after 7 days. Such evidence demonstrates that a possible oxidation process mainly affects the Cd-rich NC surface and does not influence the carrier confinement in the PbS internal area, where the band-edge radiative recombination occurs. In addition, the surface oxidation induces a surface-defect passivation, reducing the density of defects responsible for non-radiative recombination processes and contributing to the enhancement of the PL intensity.

Summarizing, spectroscopic investigation seems to indicate that the very rapid cation-exchange process reduces the PbS NC diameter from 2.3 nm of the native PbS NCs to 2.0 nm. It is worth recalling that, while CdS is a wide-gap semiconductor having emission in the blue-region of the spectrum and average PL lifetime of few nanoseconds, [62] PbS is a small-band gap semiconductor with PL in the NIR and average PL lifetime of a few microseconds. The obtained PbS–CdS NCs, characterized by a Cd excess (Cd/Pb = 1.5 from STEM-EDS), an hypothetical ternary PbCdS alloy, still in the quantum confinement regime and extended to the whole NC volume, would be expected to have spectroscopic properties very close to those of CdS, in contrast with experimental evidence [63]. XRPD analysis revealed that the NC diameters are in agreement with the values calculated from spectroscopic results, and determine rock-salt as the crystalline phase for both PbS and PbS–CdS NCs. On the other hand, HR-TEM investigation established larger sizes both for PbS and PbS–CdS NCs. However, the small size of the NCs limited the possibility to obtain precise STEM-EDS mapping, not confirming the formation and composition of an albeit thin CdS shell on the PbS surface. Following the experimental results and previous considerations, it is reasonable to describe the obtained PbS–CdS nanostructures, after the cation exchange process, with a model not too far from a core–shell system. Such nanostructures demonstrate a Cd-enriched surface, which could form a very thin CdS shell or, most probably, a PbSCd ternary alloy or a graded shell and whose extension could be not uniform on a NC surface, while preserving a reduced portion of PbS, still in the quantum confinement regime, responsible for the spectroscopic properties. Such PbS–CdS NCs absorb and emit in a spectral range at the boundary between red and NIR, a region of interest for photovoltaic, lighting and biomedical applications. The intense enhancement (about 7 times) of PL with respect to the starting PbS NCs and the increased stability of the emission over time indicates the effectiveness of the surface passivation. Such NCs have demonstrated intense emission and high chemical and optical stability over time and have been successfully exploited for the fabrication of NIR NC-LEDs [11], exhibiting a very low turn-on voltage without parasitic electroluminescence and high active area devices.

## 4. Conclusions

We reported the colloidal synthesis of very small PbS–CdS NCs by the cation exchange procedure and their structural and optical characterization. Crystallographic measurements pointed out that pristine PbS NCs had a rock-salt structure even after the cation exchange, resulting in a smaller diameter due to a lattice contraction, as Cd ions in the PbS host lattice are expected to produce. The NC size contraction was confirmed by HR-TEM analysis while STEM-EDS atomic quantification evidenced an excess of Cd in PbS–CdS NCs. Spectroscopic results revealed that, during the cation exchange process, a passivation layer effectively grows at the expense of the native PbS, resulting in smaller NCs characterized by a brighter blue-shifted PL in the border region between red and NIR, a region of interest for photovoltaic, lighting and biomedical applications. Such a Cd enriched surface limits the non-radiative recombination processes occurring at surface trap-sites, also resulting in slower average excited state lifetimes. Due to the small size of NCs, the structural observations do not confirm the formation of a pure CdS shell on the PbS–CdS surface; however, the spectroscopic results endorsed the formation of a nanostructure in which a reduced PbS portion is responsible for the emission properties. The intense enhancement (about 7 times) of PL with respect to the starting PbS NCs, and the increased stability of the emission over time, indicates the effectiveness of the surface passivation.
